# An Actin-Based Wave Generator Organizes Cell Motility

**DOI:** 10.1371/journal.pbio.0050221

**Published:** 2007-08-14

**Authors:** Orion D Weiner, William A Marganski, Lani F Wu, Steven J Altschuler, Marc W Kirschner

**Affiliations:** 1 Department of Biochemistry, University of California San Francisco, San Francisco, California, United States of America; 2 Cardiovascular Research Institute, University of California San Francisco, San Francisco, California, United States of America; 3 Department of Systems Biology, Harvard Medical School, Boston, Massachusetts, United States of America; 4 Department of Pharmacology, Southwestern Medical School, Dallas, Texas, United States of America; 5 Green Comprehensive Center for Molecular, Computational, and Systems Biology, Southwestern Medical School, Dallas, Texas, United States of America; Adolf-Butenandt-Institut, Germany

## Abstract

Although many of the regulators of actin assembly are known, we do not understand how these components act together to organize cell shape and movement. To address this question, we analyzed the spatial dynamics of a key actin regulator—the Scar/WAVE complex—which plays an important role in regulating cell shape in both metazoans and plants. We have recently discovered that the Hem-1/Nap1 component of the Scar/WAVE complex localizes to propagating waves that appear to organize the leading edge of a motile immune cell, the human neutrophil. Actin is both an output and input to the Scar/WAVE complex: the complex stimulates actin assembly, and actin polymer is also required to remove the complex from the membrane. These reciprocal interactions appear to generate propagated waves of actin nucleation that exhibit many of the properties of morphogenesis in motile cells, such as the ability of cells to flow around barriers and the intricate spatial organization of protrusion at the leading edge. We propose that cell motility results from the collective behavior of multiple self-organizing waves.

## Author Summary

Many cells guide their movement in response to external cues. This ability is required for single-celled organisms to hunt and mate, enables innate immune cells to seek and destroy pathogens, and is also essential for the development of multicellular organisms. Misregulation of cell migration is intimately involved in atherosclerosis and in cancer metastasis. Although many of the regulators of cell migration are known, we do not understand how these components act together to organize cell shape and movement. We used advanced light microscopy to follow the distribution of a key regulator of cell migration in living cells. We focus on a protein called Hem-1, which is part of a large multimolecular protein complex that regulates cell shape in animals and plants. We found that Hem-1 exhibits complex cycles of activation and inhibition to generate waves of propagating Hem-1 and actin assembly that are similar in mechanism to grass fires or the action potentials used in neuronal signaling. These waves potentially explain many of the complex behaviors of motile cells such as the ability of cells to flow around barriers and the intricate spatial organization of protrusion at the front of moving cells.

## Abstract 3

Reciprocal interactions between actin and the Scar/WAVE complex appear to generate propagated waves of actin nucleation that embody many of the properties of morphogenesis in motile cells.

## Introduction

Rac and its downstream effector Scar/WAVE are central regulators of cell shape and movement [1–4]. Rac binds WAVE indirectly via a multiprotein complex [5]. This complex contains the WAVE protein, which activates the actin nucleating Arp2/3 complex, and four other proteins that may regulate or localize the complex. The Hem-1 component (homologous to Nap1) of the leukocyte WAVE2 complex is required for actin polymerization and proper leading edge morphology in neutrophils [6], and its homologues play important roles in regulating cell shape or movement for *Dictyostelium* [7], plants [8], worms [9], insects [10–12], and mammals [1,13,14].

Although many of the regulators of actin assembly are known, understanding how they act together to generate cell morphology is a much more difficult problem. Spatial and dynamic information will be key to understanding their overall function. We show here that the leading edge of neutrophils contains moving waves of Hem-1 (including complexes which contain WAVE2), whose collective behavior corresponds to the morphology of the leading edge. These waves are not formed by lateral movement of individual proteins but, like action potentials, are the result of propagated cycles of activation and inhibition. These waves reveal a far more complex and dynamic interaction between inducers of actin nucleation and the cytoskeleton than is represented in current models of cell motility.

## Results

### The Hem-1/Nap1 Component of the WAVE2 Complex Localizes to Propagating Waves

To analyze spatial and temporal dynamics of signaling and morphogenesis, we and others have used confocal or wide-field microscopy of fluorescently tagged proteins. During polarization and migration, some proteins localize to the leading edge, others to the trailing edge, and some appear uniform [15–21].

**Figure 1:**
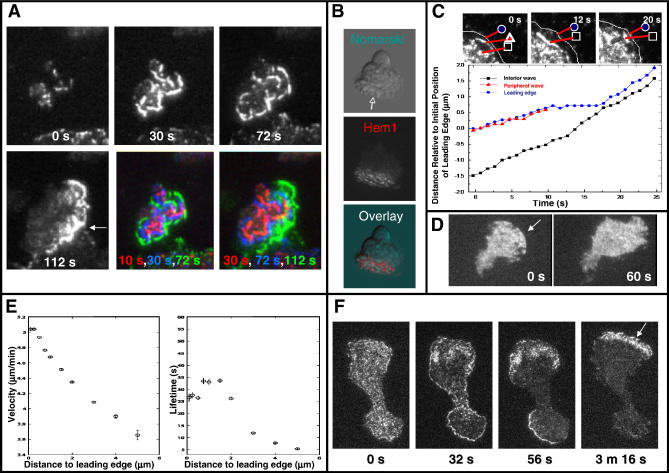
Hem-1 Component of the WAVE2 Complex Localizes to Multiple Propagating Waves in Chemoattractant-Stimulated HL-60 Cells (A) HL-60 cells expressing Hem-1-YFP continually exposed to chemoattractant (20 nM fMLP)—see Video S1 and Video S2. Hem-1 initially concentrates in foci, which form outwardly propagating waves that eventually develop into a polarized accumulation of Hem-1 at the leading edge (denoted by arrow at 112 s). Panels five and six overlay successive Hem-1 distributions in red, blue, and green successively. (B) Hem-1 waves concentrate at the ruffled leading edge of polarized cells (leading edge identified by arrow in Nomarski panel). Nomarski (Video S3), Hem-1-YFP (Video S4), and overlay (Video S5) of polarized HL-60 cell exposed to 20nM fMLP. (C) Leading edge advance is highly correlated with underlying Hem-1 waves. The top panel shows position of leading edge (blue circles and white cell outline), most peripheral Hem-1 wave (red triangles), and more interior Hem-1 wave (black squares). As the peripheral wave is extinguished, the leading edge stalls and resumes movement once the interior wave approaches the leading edge. The lower panel displays distances relative to initial position of leading edge. (D) TIRF membrane control. C5A receptor-GFP (which is uniformly distributed on the plasma membrane) is uniformly distributed in the TIRF field. An arrow in the first panel denotes the leading edge. (E) Hem-1 waves exhibit a spatial gradient in velocity (first panel) and lifetime (second panel) with respect to the leading edge. (F) HL-60 cells acutely stimulated with chemoattractant (20 nM fMLP) produce a uniform field of Hem-1 spots, which asymmetrically disappear, are retained at one end of the cell (arrow), and begin generating Hem-1 waves (Video S7).

We have analyzed some of the same components by using total internal reflection fluorescence (TIRF) microscopy, which, with its considerably higher resolution in the vertical axis, is especially useful for visualizing events at the plasma membrane. By confocal microscopy, we found that Hem-1-GFP, which assembles into the WAVE complex and other multiprotein complexes, exhibits a nearly uniform leading-edge accumulation [6]. In contrast, Hem-1-YFP visualized by TIRF exhibits much more complex local patterns (Figure 1). Hem-1 initially accumulates on the membrane as foci, which burst into outwardly propagating waves (Figure 1A and Videos S1 and S2. Note that here and throughout the rest of this manuscript, the supplemental videos capture the dynamics much more clearly than a few individual frames). In polarized cells, Hem-1 waves are concentrated near the leading edge (Figure 1A, arrow) but are not confined to the cell periphery (Figure 1A and 1B and Video S5). The morphology (Figure 1B) and speed (Figure 1C) of leading edge advance is highly correlated with the most peripheral Hem-1 waves. The complex structure of the leading edge is composed of many discrete waves (Figure 1A–1C).

Two lines of evidence suggest that these waves are not an artifact of the narrow depth of field of TIRF. First, in TIRF microscopy, proteins that are expressed uniformly throughout the cell membrane (such as the C5a receptor) show no wavelike behavior and appear uniform (Figure 1D), indicating that Hem-1 waves do not represent portions of the plasma membrane coming in and out of the very thin TIRF illumination field. Second, with very careful critical focusing, the Hem-1 waves can be visualized satisfactorily by confocal microscopy, which has a depth of field about 5–10 times larger than that of TIRF (Video S6). Hem-1 is observed in regions of the plasma membrane other than the ventral surface of the cell, but we do not have the signal-to-noise ratio necessary to determine whether Hem-1 is wavelike in these regions [6].

### Hem-1 Wave Distribution, Velocity, and Lifetime Are Nonuniform in Polarized Cells

In neutrophils polarized by exposure to a chemotactic signal, Hem-1 waves are nonuniformly distributed and have different dynamics in different regions of the cell. These features suggest an underlying regulator of Hem-1 waves that itself would be spatially polarized. The velocity of the Hem-1 waves varies reproducibly with the distance from the leading edge (5 μm/min at the leading edge versus 3.6 μm/min at 5 μm from the leading edge [Figure 1E, first panel]; also note from Figure 1C, lower panel, that individual waves increase velocity as they approach the leading edge). There is also a strong gradient in Hem-1 wave lifetime that is inversely dependent on distance to the leading edge (34 s at 1 μm from the leading edge versus 5 s at 5 μm from the leading edge [Figure 1E, second panel]). Acute stimulation with chemotactic peptide first produces a uniform field of Hem-1 spots. These break symmetry, polarize to one end of the cell (Figure 1F, arrow), and begin generating Hem-1 waves at the leading edge (Figure 1F, Video S7).

### Rac Activity and the Arp2/3 Complex Spatially Correlate with but Are More Diffuse than Hem-1 Waves

**Figure 2:**
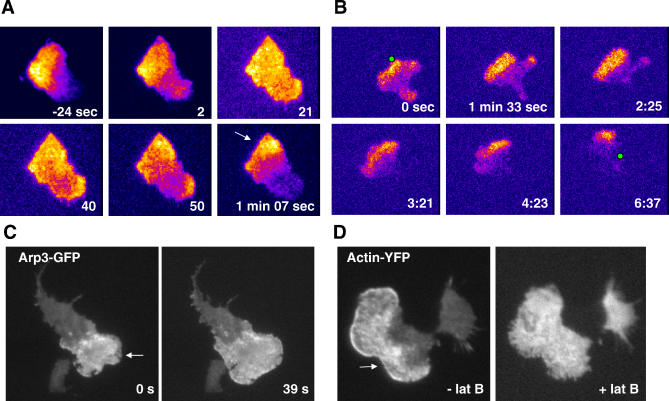
Rac Activity and the Arp2/3 Complex Spatially Correlate with but Are more Homogeneous than Hem-1 Waves (A) Pak-GBD-YFP (Rac activity probe) dynamics during acute cell stimulation (chemoattractant added to pre-polarized cell at *t* = 0 s)—see Video S8. Rac activation is initially uniform and then polarizes (arrow), similar to the spatial dynamics of Hem-1 waves (compare to Figure 4B). Rac exhibits relatively homogeneous zones of activation in contrast to discrete points and waves of Hem-1. (B) Rac activity dynamics during cell migration (see Video S9). Rac-GTP (assayed by Pak-GBD-YFP) concentrates at the leading edge (and dominant pseudopod when multiple pseudopodia are present) in cells migrating in uniform chemoattractant. Green dots are included in first and last frame as fiduciary marks to clarify relative cell positions. (C) The Arp3-GFP component of the Arp2/3 complex fills the leading edge (arrow) and exhibits a more homogeneous distribution than Hem-1 waves (Video S10). The cell is representative of more than five similar cells. (D) Actin-YFP is more homogeneously distributed than Hem-1 waves throughout the leading edge of polarized cells. Latrunculin treatment of actin-YFP–expressing cell abolishes leading edge accumulation of actin-YFP. Cells are representative of more than five similar cells.

What could account for the polarized distributions, velocities, and lifetimes of Hem-1 waves in moving cells? Active Rac is a likely candidate, because it directly binds the WAVE2 complex (thereby indirectly binding Hem-1), and the WAVE2 complex is required for Rac-induced actin assembly [1]. To visualize the spatial dynamics of Rac activation (Rac-GTP) in motile cells, two basic approaches have been used in the past. The first relies on fluorescence resonance energy transfer between a fluorescently tagged Rac GTPase and a tagged effector that binds activated Rac (Pak GTPase binding domain [GBD]) [22,23]. Because Rac is untagged, it is not possible to visualize activation of endogenous Rac with this approach. A complementary approach relies on fluorescently-tagged Pak-GBD–green fluorescent protein (GFP) to visualize activation of endogenous Rac, but this technique has only been successfully applied to fixed permeabilized cells [24]. With the significantly increased signal-to-noise of TIRF microscopy, we are now able to visualize the activation of endogenous Rac in living cells using Pak-GBD–yellow fluorescent protein (YFP) (Figure 2A and 2B. Note that previous experiments indicate that this probe is more sensitive to Rac-GTP levels than Cdc42-GTP levels in HL-60 cells [24]).

**Figure 3:**
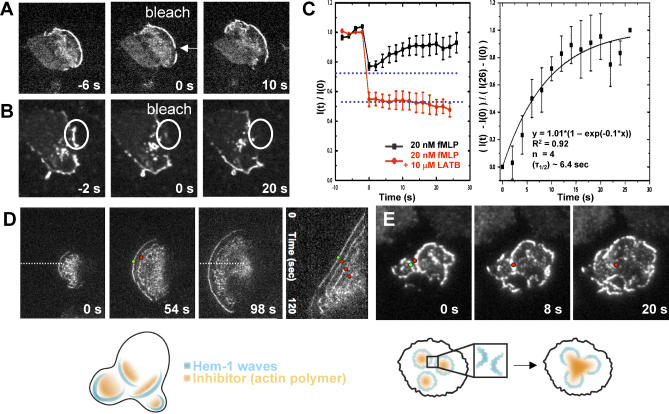
Hem-1 Waves Result from Propagated Recruitment and Release of Hem-1, Not a Moving Front of Translocated Protein (A) Control Hem-1-YFP–expressing cells (pre-polarized in uniform chemoattractant) rapidly recover fluorescence intensity following photobleaching, indicating rapid exchange of membrane-bound Hem-1 with the cytosolic pool (Video S11). (B) Depolymerization of the actin cytoskeleton with latrunculin inhibits the recovery of Hem-1-YFP fluorescence intensity following photobleaching indicating a lack of exchange of Hem-1 in the absence of actin polymers. Cells were first stimulated with uniform fMLP, treated with latrunculin until the intensity of membrane-bound Hem-1 stabilized (greater than 3 min), and then a 1–2-μm radius spot was photobleached. (C) Quantitation of Hem-1-YFP intensity following photobleaching of control (black, *n* = 4 cells) or latrunculin-treated cells (red, *n* = 5 cells). The right panel represents fit of control cell Hem-1 FRAP data (half-life on membrane = 6.4 s, obtained from four cells). (D) Cell with single dominant peripheral Hem-1 wave and multiple interior waves shows a clear refractory region between the most peripheral Hem-1 wave (green dot) and the next wave (red dot)—see Video S12. Final panel is kymograph of the same cell. Vertical axis of the kymograph is time and horizontal axis is spatial position corresponding to dotted line in first and third panels. The kymograph shows several sets of waves (leading edge green dot, subsequent waves red dots) with clear gaps between them. The bottom schematic indicates our working model— Hem-1 waves (blue) deposit an inhibitor in their wake (orange) that transiently inhibits Hem-1 recruitment. We later present data that actin polymer represents a component of this inhibitor. (E) Annihilation of two colliding Hem-1 wavefronts (one green dot, one red dot at *t* = 0) in single cell (Video S13). Waves collide at 8 s (red dot) and are both extinguished by 20 s (red dot). The bottom schematic indicates our interpretation of colliding waves. Hem-1 waves (blue) deposit an inhibitor in their wake (orange), leading to annihilation of colliding waves. This property could enable cells to focus wave propagation and actin polymerization toward the cell periphery.

**Figure 4:**
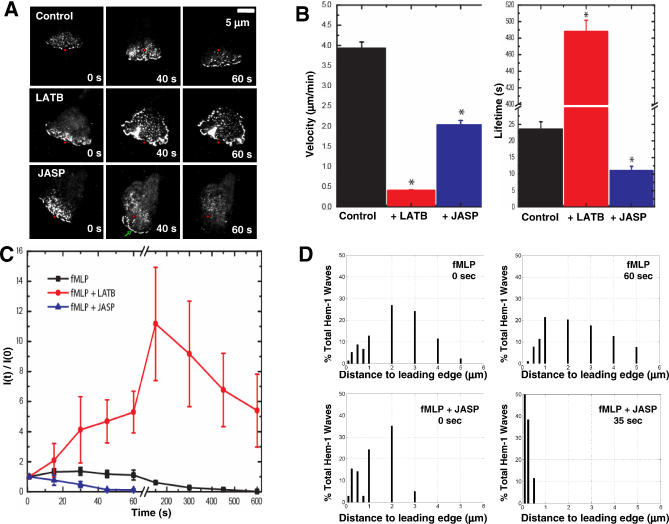
Actin Polymers Are Required for Wave Movement and Hem-1 Recycling (A) Hem-1 waves in control cells (Video S14), cells treated with latrunculin (which sequesters actin monomers, leading to depolymerization of the actin cytoskeleton, Video S15 and S16), or cells treated with jasplakinolide (which stabilizes actin filaments against disassembly, Video S17). All cells were allowed to polarize in response to 20 nM fMLP prior to drug treatment. Red dots are included in all panels as fiduciary marks to clarify relative cell positions between frames. Green arrow at 40 s time point for jasplakinolide-treated cell indicates Hem-1 waves at cell periphery with no significant interior waves. (B) Quantitation of Hem-1 wave velocity (left panel) and lifetime (right panel) for control and drug-treated cells. *n* = 5–8 cells for each condition for (B–D). (C) Quantitation of Hem-1 wave intensity relative to *t* = 0 for control and drug-treated cells. (D) Histogram of Hem-1 wave distribution at two different time points in control or jasplakinolide-treated cells.

In contrast to the discrete waves of Hem-1, cells exhibit more homogeneous zones of Rac activation (Figure 2A and 2B, Videos S9 and S10). The spatial distribution of Rac activation generally overlaps with the portion of the cell containing Hem-1 waves for cells acutely stimulated with chemoattractant (compare Figure 2A with Figure 1F). Rac activation also correlates with Hem-1 wave distribution during cell migration (compare Figure 2B with Figure 1A and 1B). However, the zones of Rac activation are homogeneous, whereas Hem-1 forms sharp wavefronts, suggesting that Hem-1 does not simply mirror the presence of active Rac on the membrane, which instead may provide a permissive condition for Hem-1 waves to form.

What is the spatial distribution of the downstream effectors of Hem-1 waves? The WAVE2 component of Hem-1 complexes is thought to act through the Arp2/3 complex to induce actin assembly. WAVE directly activates the Arp2/3 complex [25], and WAVE complex mutants phenocopy loss of the Arp2/3 complex in a variety of systems [9,26–29]. In contrast to Hem-1, the Arp2/3 complex (Figure 2C and Video S10) and total actin (Figure 2D) fill the leading edge of chemoattractant-stimulated neutrophils without discrete gaps, possibly because Arp2/3 complex (which incorporates into polymerized actin) and actin polymer represent a history of actin assembly versus the current sites of actin nucleation.

### The Waves Result from Propagated Recruitment and Release of Hem-1; the Waves Are Not a Moving Front of Translocated Protein

Do propagated Hem-1 waves represent sequential rounds of protein recruitment and release from the soluble pool, or do they represent the movement of Hem-1 proteins in the plane of the membrane, powered by actin polymerization, motor proteins, or some other process? To distinguish between these very different mechanisms, we photobleached a segment of an advancing wave (Figure 3A–3C). Lack of recovery after photobleaching and maintenance of the bleached spot as the rest of the wave moves outward would indicate that a wave is composed of a nonexchangeable pool of Hem-1 moving in the plane of the membrane. In contrast, the photobleached Hem-1 gap very rapidly recovers before there is appreciable forward progress of the wave, suggesting that the recruited Hem-1 protein dynamically equilibrates with the cytosolic pool and that the bulk of wave movement is generated by recruiting more Hem-1 from the cytosol (Figure 3A and 3C, Video S11). Recruited Hem-1 only transiently associates with the membrane (half life *t*_1/2_ = 6.4 s, Figure 3C, second panel). Hem-1 recruitment occurs with high probability adjacent to existing membrane-bound Hem-1 (99% ± 1% of newly-observed Hem-1 is observed within 0.2 μm of existing Hem-1 foci and 0.15% ± .1% is observed greater than 0.2 μm away. The distribution that would be expected for random Hem-1 association with the membrane would be 50.3% ± 14% within 0.2 μm of existing Hem-1 foci and 48.5% ± 13% more than 0.2 μm from existing Hem-1 foci).

Actin polymer is required for the rapid equilibration of Hem-1 with the cytoplasmic pool. When latrunculin is added to depolymerize filamentous actin, the photobleached Hem-1 spot is stable for at least 10 min, after which the cells detach from the substrate (Figure 3B). Collectively, these data make two important points. First, Hem-1 waves move by sequential rounds of protein recruitment, not by a significant amount of movement of individual Hem-1 complexes in the plane of the membrane. Second, the observed rapid recycling of recruited Hem-1 depends on polymeric actin.

### Hem-1 Wavefronts Generate Zones of Inhibition and Annihilate upon Collision

Particularly in cells with a single strong peripheral Hem-1 wave and multiple interior waves, a gap between waves can be easily observed, presumably reflecting an inhibitory period between waves (Figure 3D, Video S12). When other biological waves collide, they annihilate, because the region in the wake of each wave is transiently inhibitory to further activation [30,31]. In a similar fashion, colliding Hem-1 waves also annihilate (Figure 3E, Video S13). Annihilation of colliding waves could potentially account for the observations that peripheral waves are more continuous than internal waves and that waves tend to propagate toward the cell periphery (Figures 1 and 3).

### Actin Polymer Is Required for Wave Movement and Hem-1 Recycling

What downstream products of Hem-1 complex recruitment might act to inhibit further Hem-1 recruitment between waves? Actin polymer is a likely candidate, because actin nucleation is a known output of the WAVE2 complex [1], and polymeric actin fills the leading edge (Figure 2D). To test a role for actin as an inhibitor of further Hem-1 complex recruitment, we exposed cells to drugs that either stabilize or destabilize the actin cytoskeleton. In contrast to fibroblasts, which require actin polymers and adhesion for signal transduction downstream of Rac [32], neutrophils require neither adhesion nor actin polymer for activating Rac effectors [6], making perturbations of the actin cytoskeleton less confounding for neutrophils.

In untreated cells, waves propagate with a characteristic velocity of 3.93 ± 0.15 μm/min and persist with average lifetime of 23.52 ±- 2.24 s, (Figure 4A and 4B, Video S14). Depolymerization of actin with latrunculin increases wave lifetime 20-fold to 488 ± 13.6 s and increases the intensity of membrane-associated Hem-1 by more than an order of magnitude, suggesting that actin polymer drives Hem-1 off of the membrane (Figure 4B and 4C, Videos S15 and S16). These data are consistent with the lack of exchange of membrane-associated Hem-1 following actin depolymerization (Figure 3B and 3C). Latrunculin reduces wave motility 10-fold to 0.41 ± 0.02 μm/min (Figure 4A and 4B); the residual motion is nondirectional and most likely represents random diffusion. In contrast, jasplakinolide, which stabilizes actin polymers against depolymerization, produces a phenotype that is consistent with an enlarged field of inhibition between waves (Figure 4A and 4D, Video S17). In the presence of jasplakinolide, the wave closest to the leading edge initially persists upon actin stabilization (Figure 4A, green arrow), but interior waves are extinguished (Figure 4A, 40 s time point, and Figure 4D). The stabilization of the actin cytoskeleton produced by jasplakinolide decreases the velocity (2.04 ± 0.11 μm /min), intensity, and lifetime (11 ± 1.27 s) of the remaining Hem-1 waves (Figure 4B and 4C). These data suggest that actin polymer is required for Hem-1 wave movement as well as the removal of Hem-1 from the plasma membrane.

### Hem-1 Waves Are Extinguished upon Contact with Mechanical Barriers

**Figure 5:**
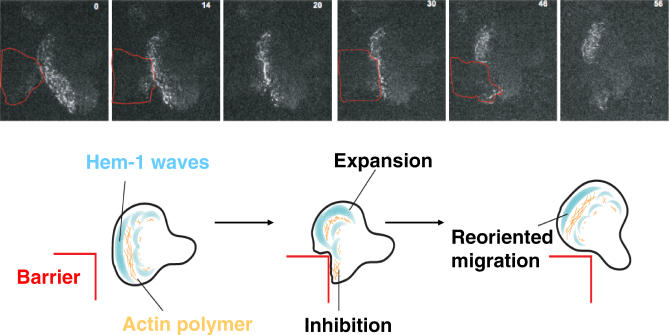
Hem-1 Waves Extinguish at Mechanical Barriers Hem-1 waves that collide with boundary (cell outlined in red) are extinguished, whereas those that do not continue to propagate (Video S18). The bottom schematic depicts our hypothesis for barrier avoidance. Hem-1 waves normally propagate just ahead of the actin inhibitory field. Stalling of waves by external barriers prevents Hem-1 from escaping removal by actin polymer-dependent inhibitory process, causing these waves to be extinguished. Waves in other regions of the cell continue to expand, enabling the cell to flow around barriers.

As cells migrate, Hem-1 waves propagate with a characteristic velocity and lifetime toward the cell periphery (Figure 1E). We never observe the Hem-1 waves to stall for more than a few seconds except when actin is depolymerized. We believe this is because a standing wave of Hem-1 on the membrane would be destabilized by locally generated actin polymers (Figures 3 and 4). However, stalling can be forced to occur when a cell encounters a physical barrier that prevents further membrane protrusion. In such a condition, the Hem-1 waves stall and are extinguished (Figure 5, Video S18). When this happens, other regions of the cell that are not in contact with the external barrier continue to propagate Hem-1 waves and expand in size. The effect of this behavior is that the cell has effectively restricted actin polymerization and leading edge organization to domains compatible with productive movement, potentially accounting for contact inhibition of movement. As a result, rather than be stymied by physical barriers, cells tend to flow around them. The integration of signaling and actin polymerization in Hem-1 waves ensures that the cytoskeleton is not simply a passive readout of the cell polarity but rather has local dynamic properties of its own that contribute to the overall behavior and give it some of its most life-like properties.

## Discussion

### A Heuristic Model for the Hem-1 Wave Generator

**Figure 6:**
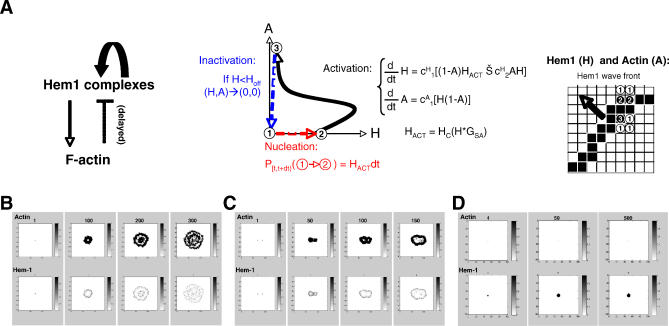
Simulations of Hem-1 Wave Generator (A) Outline of biological model and mathematical simulations. The Hem-1 wave circuit is wired similarly to other biological waves such as action potentials and relies on autoactivation (Hem-1 recruitment adjacent to existing Hem-1 distributions, which is observed 99% ± 1% of the time in living cells) and delayed inhibition (actin polymers inhibiting Hem-1 membrane association). Membrane-associated Hem-1 locally generates actin polymers via the WAVE2 complex, and more Hem-1 is recruited. After a delay, actin polymers locally remove Hem-1 from the membrane. The process repeats until old actin polymers are ultimately broken down, and the process continues. The lifetime of the actin polymers dictates the length of the gaps between waves. The Matlab code used for Figure 6 is presented in Protocol S1. Our mathematical simulations are based on the parameters listed in Table 1. (B) Simulations of polarity circuit in Figure 6A (Video S19). Combining reciprocal interactions of Hem-1 waves and actin produces Hem-1 waves that lead actin assembly and move with a characteristic gap between waves (roughly corresponding with lifetime of actin polymer). The top panel represents actin polymer, and the bottom panel represents membrane-associated Hem-1. (C) Simulation of colliding waves based on model in Figure 6A (Video S20). The top panel represents actin polymer, and the bottom panel represents membrane-associated Hem-1. Colliding waves annihilate. Compare with colliding Hem-1 waves in living cell Figure 3E and Video S13. (D) Actin depolymerization freezes waves in simulations. The simulation is based on model in Figure 6A (Video S21). Depolymerization of the actin cytoskeleton (by setting 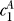 = 0) freezes Hem-1 waves and increases their intensity, mirroring the behavior of living cells (compare with Figure 4A, Videos S15 and S16). The top panel represents actin polymer, and the bottom panel represents membrane-associated Hem-1.

Based on the observed in vivo dynamics of Hem-1, we propose a model for Hem-1 waves that is similar to the circuitry of other biological waves, such as action potentials (Figure 6A, first panel). Such biological waves are based on autoactivation and delayed inhibition. Our photobleaching experiments (Figure 3A–3C) suggest that Hem-1 waves result from successive rounds of Hem-1 recruitment and release. Consistent with this idea, Hem-1 recruitment is observed with high probability adjacent to existing Hem-1 membrane distributions. Several pieces of evidence suggest that the actin polymerization generated downstream of Hem-1 complexes is required to remove Hem-1 from the membrane (potentially forming the autoinhibitory portion of the cycle). First, Hem-1 waves normally have characteristic lifetime and intensity, and actin depolymerization increases both more than an order of magnitude (Figure 4B and 4C). Second, Hem-1 normally cycles on and off the membrane, and actin depolymerization stops this flux (Figure 3A–3C). Third, there is a gap observed between Hem-1 waves (Figure 3D), and stabilization of the actin cytoskeleton increases the length of this gap (Figure 4D). Fourth, stabilization of the actin cytoskeleton decreases the velocity, intensity, and lifetime of Hem-1 waves (Figure 4B and 4C).

**Table 1:**
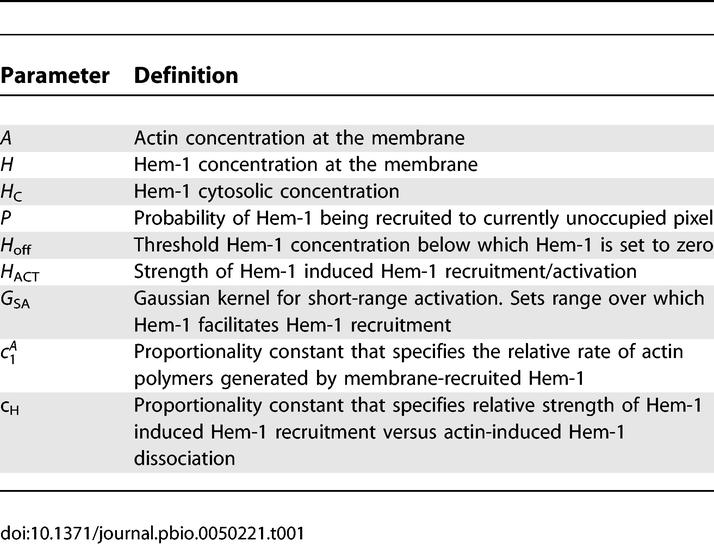
Parameters for Mathematical Simulations of Hem-1 Waves

Using mathematical simulation, we asked whether a simple circuit involving auto-activation and transient inhibition could generate Hem-1 waves with the properties we observed (Figure 6, Video S19–S21). These simulations (done under a chosen set of parameters, not experimentally confirmed, and generally not experimentally accessible at this time) recapitulate most of the basic behaviors of Hem-1 waves (Figure 6B and 6C). They indicate that Hem-1 wave movement as well as Hem-1 removal can be explained simply by the inhibitory role of actin polymers for Hem-1 membrane association. In the simulations, actin depolymerization not only increases Hem-1 wave intensity and lifetime but also stops wave movement due to depletion of cytosolic Hem-1 (Figure 6D, Video S21). Consistent with this, we observe biochemically that the majority of Hem-1 translocates to the plasma membrane in response to latrunculin treatment (unpublished data). The propagated waves occur over a range of parameters where actin polymers are metastable, as they are in the cell (Figure S1). However, additional components almost certainly contribute to wave organization, and specific experimental parameters will need to be evaluated. Yet this basic circuit indicates that these propagated waves may be based on simple and general properties of the system.

Oscillatory and wavelike patterns for both actin polymers and the Arp2/3 complex have been observed in *Dictyostelium* and in other systems [33–36]. Furthermore, wavelike protrusive activity has been noted from *Drosophila* cells to mammalian cells [37]. These activities exhibit different organization from the Hem-1 waves, although they may be related to them. Conceptually, however, there is a strong distinction between actin and the Arp2/3 complex, which are incorporated into the cytoskeleton and represent the physical substrate that generates protrusion, and the Hem-1 waves that represent a moving inductive field that patterns this substrate. When actin is depolymerized, it and the Arp2/3 complex dissociate from the cortex, whereas Hem-1 continues to accumulate at the membrane. Yet the behavior of the Hem-1 waves is closely tied to the actin substrate, and this, in turn, produces a moving front of actin nucleation. These data suggest a different view of cell motility as neither a global process at the level of whole cell organization nor a biasing of local actin polymerization events organized at the molecular level. Rather, cell movement may result from the collective behavior of multiple self-organizing waves, an idea consistent with the morphological pattern of leukocyte movement during chemotaxis [38]. The wavelike organization of protrusion has been observed in cells from flies to mammals [37], and the WAVE complex plays a conserved role in morphogenesis throughout plants and metazoans [6–14]. We propose that these Hem-1/Nap1 waves may also occur in many cell types and serve as a conserved subcircuit for cellular morphogenesis and movement.

## Materials and Methods

### Reagents.

The following reagents were used: Latrunculin (Calbiochem; http://www.emdbiosciences.com/html/CBC/home.html), jasplakinolide (Calbiochem), formyl met-leu-phe (fMLP; Sigma; http://www.sigmaaldrich.com), low endotoxin human serum albumin (Sigma), and human fibronectin (Sigma).

### Cell lines.

HL-60 cell stably expressing Hem-1-YFP, C5AR-GFP, and actin-YFP were generated as previously described [6,39,40]. Arp3-GFP cells were generated by retroviral-based infection of HL-60 cells using the pLNCX vector and GPG packaging cell lines [41]. Stable cell lines were obtained after neomycin selection.

Cells were cultured and differentiated as previously described [39]. Cells were plated on coverslips precoated with 0.2 mg/ml human fibronectin, and cells were stimulated with 20 nM fMLP in modified Hank's buffered saline solution (mHBSS) containing 0.2% human serum albumin. For drug treatments, cells were either simultaneously treated with drug and stimulated with fMLP or pretreated with drugs prior to exposure to fMLP. All experiments were performed at room temperature.

### Imaging/data analysis.

TIRF microscopy images were acquired on a Nikon TE2000E2 Inverted microscope equipped with a 1.45 numerical aperture (NA) 60× and 100× PlanApo TIRF objectives and an electron microscopy charge coupled device (EM-CCD) (Hamamatsu; http://www.hamamatsu.com/ or Roper 512 II; http://www.roperscientific.com/). Typical imaging conditions for most experiments used 100 ms exposures every 1–2 s with 20 mW 488 nm or 514 nm laser lines attenuated 8–64-fold with neutral density filters, and near maximal multiplication on the intensified CCDs to minimize phototoxicity and photobleaching (these cells are very sensitive to phototoxicity). For some experiments, a laser-based autofocus system (Perfect Focus, Nikon; http://www.nikonusa.com) was used to minimize sample drift. Metamorph or Nikon Elements were used for image acquisition. ImageJ was used to generate kymographs and pseudocolored images.

### Measurement of Hem-1 particle velocity and lifetime.

All image analysis was performed using Matlab 7.0.4 (http://www.mathworks.com)and the Image Processing Toolbox 5.0.2. Sequential TIRF images of a single cell expressing fluorescent Hem-1 were acquired and then the image set was processed in the following manner. First, the images were segmented with a user-defined threshold to separate out the background from the regions where Hem-1 particles were expressed. Second, the displacement of each particle between consecutive image frames was computed using a correlation-based approach [42]. The displacement measurements were refined by quadratic interpolation to achieve subpixel resolution (accuracy ∼ 0.01 μm). Third, the displacements were used to follow the trajectory of each particle and to determine particle lifetime. Fourth, because the particle trajectory varied linearly with time, the average velocity of each particle during its lifetime was measured with curve fitting. Fifth, the position of the cell's leading edge in each image frame was manually determined, and the distance between the leading edge and each particle was computed.

### Assessment of Hem-1 mobility.

The mobility of Hem-1 was investigated using fluorescence recovery after photobleaching (FRAP). The Hem-1 fluorescence in a small region (∼1–2 μm in radius or a diffraction-limited spot for some experiments) at the leading edge of a chemoattractant-stimulated cell was photobleached and allowed to recover for up to 60 s. The dependence of Hem-1 mobility on actin dynamics was also investigated by photobleaching Hem-1 expressing cells that were first activated with 20 nM fMLP and then treated with 10 μM latrunculin B. The halftime of Hem-1 recovery (i.e., the time from the bleach to the time where the fluorescence intensity reaches half of the final recovered intensity) was determined by plotting the recovery of relative fluorescence within the bleached region as a function of time and fitting the data with an exponential function [43].

### Modeling and simulations.

Simulations of the Hem-1/actin circuit were performed using the Ordinary Differential Equations stochastic simulator in Matlab. Details of the code and variation of parameters used can be found in Protocol S1.

### Rationale for model and simulations.

Many of the interactions and rate constants required for an exact model of the Hem-1 waves are unknown. Hence, we chose a modeling strategy that accomplished the following: (1) directly incorporates the phenomenology experimentally observed behaviors, (2) favors the interpretability of a small number of equations and interactions over the flexibility of more complex models, and (3) will allow future refinement as more network details are clarified.

The model assumes Hem-1 and actin are present either on a membrane (represented as a grid or pixels) or in a cytosolic pool. These are represented at a pixel and time (*x*, *t*) as:

*A* is the proportion of polymerized actin at each pixel and (1 − *A*) is the proportion of unpolymerized actin at each pixel; *H* is the Hem-1 density at each pixel and *H*_C_ is the total cytosolic pool of Hem-1.

The model of Hem-1–actin interaction described in the main text is:

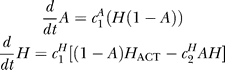



Each term is described below. Note that * indicates the mathematical convolution operation, that is: 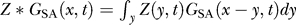 .

We modeled molecular interaction at one spatial scale (Figure 6A).

Short range Hem-1 autoactivation is modeled by convolving Hem-1 with a Gaussian kernel *G*_SA_ of small (measured in units of pixels) variance: *H*_ACT_ *= H*_C_(H * G_SA_). This term is positive when there is both available cytosolic Hem-1 and “near-by” Hem-1 (for most simulations Hem-1 at adjacent pixel).

Putting this together, in the equations above: *A*(*x*, *t*) = 0 initially, but is allowed to nucleate with probability proportionate to *H*_ACT_. Upon nucleation, actin concentration is set to a small number, and driven positive at each pixel proportionately to the amount of Hem-1 and remaining available unpolymerized actin. We assume that if Hem-1 ever falls below a (small) threshold, actin concentration is reset to zero until the next nucleation event (Figure 6A).

Hem-1 is increased proportionately to the amount of free actin and *H*_ACT_ and decreased proportionately to the amount of polymerized actin and Hem-1. Thus, if actin is fully polymerized in the pixel, the Hem-1 concentration can no longer increase. Conversely, if actin is fully depolymerized at a pixel, the Hem-1 concentration will monotonically increase.

## Supporting Information

Figure S1Parameter Scans of Wave SimulationsThe ability of our simulated actin/Hem-1 circuit (from Figure 6A) to generate waves was assayed for a range of 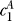 values (proportionality constant that specifies the relative rate of actin polymers generated by membrane-recruited Hem-1). The top panel indicates the average frequency between successive Hem-1 waves, and the bottom panel indicates the average Hem-1 intensity at the membrane. Multiple sets of Hem-1 waves are only observed at intermediate levels of actin assembly (red portion of top panel). At low actin assembly (as would be observed for latrunculin-treated cells), the simulations yield bright, nonmoving Hem-1 accumulations on the membrane (see Figure 6D). At high actin assembly (as would be observed for jasplakinolide-treated cells), the simulations fail to produce multiple Hem-1 waves (as in Figure 4D). Thus, the propagated waves occur over a range of parameters where actin polymers are metastable, as they are in the cell.
(29 KB PDF)

Protocol S1 Matlab Code (59 KB DOC)

Video S1 Hem-1 Waves HL-60 cells expressing Hem-1-YFP continually exposed to chemoattractant (20 nM fMLP) imaged with total internal reflection microscopy with 1 frame every 2 s. Hem-1 initially concentrates in foci, which form outwardly propagating waves that eventually develop into a polarized accumulation of Hem-1 at the leading edge. This video corresponds to the field from which the cell for Figure 1A was obtained.
(5.1 MB AVI)

Video S2 Additional Example of Hem-1 Waves HL-60 cells expressing Hem-1-YFP continually exposed to chemoattractant (20 nM fMLP) imaged with total internal reflection microscopy with 1 frame every 2 s. This is an additional example of Hem-1 waves with the same conditions as Video S1. This video is not represented by a figure in the text.
(7.0 MB AVI)

Video S3 Differential Interference Contrast Image of Hem-1–Expressing Cell HL-60 cell expressing Hem-1-YFP continually exposed to chemoattractant (20 nM fMLP) imaged with differential interference contrast (DIC) microscopy with 1 frame every 6 s. This video corresponds to the first panel of Figure 1B.
(1.5 MB AVI)

Video S4 Hem-1 Waves in TIRF Microscopy HL-60 cell expressing Hem-1-YFP continually exposed to chemoattractant (20 nM fMLP) imaged with total internal reflection microscopy with 1 frame every 6 s. This video corresponds to the second panel of Figure 1B.
(1.5 MB AVI)

Video S5 Overlay of Hem-1 Waves and DIC Imaging HL-60 cell expressing Hem-1-YFP continually exposed to chemoattractant (20 nM fMLP) imaged with total internal reflection microscopy (red) and DIC microscopy (cyan) with 1 frame every 6 s. This video corresponds to the third panel of Figure 1B, and it represents an overlay of Video S3 and Video S4.
(4.7 MB AVI)

Video S6 Hem-1 Waves Imaged with Spinning Disk Microscopy HL-60 cells expressing Hem-1-YFP continually exposed to chemoattractant (20 nM fMLP) imaged with spinning disk microscopy focused on substrate-bound surface of cell with 1 frame every 2 s. Waves are not as clear as in TIRF video but are still visible, indicating (in conjunction with other data such as C5AR membrane probe, Figure 1D) that Hem-1 waves do not represent TIRF imaging artifacts.
(6.9 MB AVI)

Video S7 Hem-1 Waves in Cell Acutely Stimulated with Chemoattractant HL-60 cell expressing Hem-1-YFP acutely exposed to chemoattractant (20 nM fMLP) at frame 24 and imaged with total internal reflection microscopy with 1 frame every 2 s. HL-60 cells acutely stimulated with chemoattractant produce a uniform field of Hem-1 spots, which asymmetrically disappear, are retained at one end of the cell, and begin generating Hem-1 waves. This video corresponds to Figure 1F.
(5.6 MB AVI)

Video S8 Rac Activation Dynamics during Acute Cell Stimulation HL-60 cell expressing Pak-GBD-YFP (Rac activity probe) acutely exposed to chemoattractant (20nM fMLP). Rac activation is initially uniform then polarizes, similar to the spatial dynamics of Hem-1 waves (compare to Figure 1F and Video S7). Note that Rac exhibits relatively homogeneous zones of activation in contrast to discrete points and waves of Hem-1. This video corresponds to Figure 2A.
(2.3 MB AVI)

Video S9 Rac Activation Dynamics during Cell Migration HL-60 cell expressing Pak-GBD-YFP (Rac activity probe) in the presence of uniform 20 nM fMLP. Rac-GTP concentrates at the leading edge (and dominant pseudopod when multiple pseudopodia are present) in cells migrating in uniform chemoattractant. This video corresponds to Figure 2B.
(2.9 MB AVI)

Video S10 Arp3-GFP Images with TIRF Microscopy HL-60 cell expressing Arp3-GFP continually exposed to chemoattractant (20 nM fMLP) imaged with TIRF microscopy with 1 frame every 2 s. The Arp3 component of the Arp2/3 complex fills in the leading edge and exhibits a more homogeneous distribution than Hem-1 waves. This video corresponds to Figure 2C.
(3.9 MB AVI)

Video S11 Photobleaching of Hem-1 Waves Control Hem-1-YFP–expressing cells (pre-polarized in uniform chemoattractant) rapidly recover fluorescence intensity following photobleaching, indicating rapid exchange of membrane-bound Hem-1 with the cytosolic pool. This video corresponds to Figure 3A.
(1.2 MB AVI)

Video S12 Gaps between Hem-1 Waves HL-60 cell expressing Hem-1-YFP continually exposed to chemoattractant (20 nM fMLP) imaged with total internal reflection microscopy with 1 frame every 2 s. Cell with single dominant peripheral Hem-1 wave shows clear refractory region between the most peripheral Hem-1 wave and the next interior wave. This video corresponds to Figure 3D.
(5.5 MB AVI)

Video S13 Annihilation of Colliding Hem-1 Waves HL-60 cell expressing Hem-1-YFP continually exposed to chemoattractant (20 nM fMLP) imaged with total internal reflection microscopy with 1 frame every 2 s. Hem-1 waves within a single cell annihilate upon collision. This video corresponds to Figure 3E.
(4.6 MB AVI)

Video S14 Untreated Hem-1-YFP–Expressing Cell HL-60 cell expressing Hem-1-YFP continually exposed to chemoattractant (20 nM fMLP) imaged with total internal reflection microscopy with 1 frame every 2 s. This video corresponds to the top panel of Figure 4A.
(3.7 MB AVI)

Video S15 Hem-1-YFP–Expressing Cell Treated with Actin-Destabilizing Drug HL-60 cell expressing Hem-1-YFP continually exposed to chemoattractant (20 nM fMLP) imaged with total internal reflection microscopy with 1 frame every 2 s. Hem-1 waves in cells first polarized by exposure to uniform chemoattractant then treated with latrunculin (at *t* = 0) to depolymerize actin filaments. This video corresponds to the middle panel of Figure 4A.
(3.7 MB AVI)

Video S16 Hem-1-YFP–Expressing Cell Treated with Actin-Destabilizing Drug HL-60 cell expressing Hem-1-YFP continually exposed to chemoattractant (20 nM fMLP) imaged with total internal reflection microscopy with 1 frame every 2 s. Hem-1 waves in cells first polarized by exposure to uniform chemoattractant then treated with latrunculin (at frame 60, about half-way through the video) to depolymerize actin filaments. This video does not correspond to figure in paper but better reflects behavior of cell before and after latrunculin treatment (in Video S15, the cell is treated with latrunculin at first frame).
(9.3 MB AVI)

Video S17 Hem-1-YFP–Expressing Cell Treated with Actin-Stabilizing Drug HL-60 cell expressing Hem-1-YFP continually exposed to chemoattractant (20 nM fMLP) imaged with total internal reflection microscopy with 1 frame every 2 s. Hem-1 waves in cells first polarized by exposure to uniform chemoattractant then treated with jasplakinolide (at *t* = 0) to stabilize actin filaments. This video corresponds to the bottom panel of Figure 4A.
(3.7 MB AVI)

Video S18 Hem-1 Waves Flow around Boundaries HL-60 cell expressing Hem-1-YFP continually exposed to chemoattractant (20 nM fMLP) imaged with TIRF microscopy with 1 frame every 2 s. Hem-1 waves that collide with boundary (cell at left portion of field whose position is denoted in Figure 5) are extinguished whereas those that do not continue to propagate. This video corresponds to Figure 5.
(5.7 MB AVI)

Video S19 Simulations of Polarity Circuit in Figure 6A This video is a simulation of the model in Figure 6A. Combining reciprocal interactions of Hem-1 waves and actin produces Hem-1 waves that align with one another and propagate toward cell periphery. The top panel represents actin polymer, and the bottom panel represents membrane-associated Hem-1. The video corresponds to Figure 6B. For details, see Matlab code and the Materials and Methods section: Rationale for model and simulations.
(3.0 MB AVI)

Video S20 Simulation of Colliding Waves This video is a simulation based on the model in Figure 6A. The top panel represents actin polymer, and the bottom panel represents membrane-associated Hem-1. Colliding waves annihilate as in the living-cell Video S13. The video corresponds to Figure 6C.
(3.0 MB AVI)

Video S21Actin Depolymerization Freezes Waves in SimulationsThis video is a simulation based on the model in Figure 6A. Depolymerization of the actin cytoskeleton (by setting 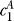 = 0) freezes Hem-1 waves and increases their intensity, mirroring the behavior of living cells (Videos S15 and S16). The top panel represents actin polymer, and the bottom panel represents membrane-associated Hem-1. The video corresponds to Figure 6D.
(2.2 MB AVI)

## Abbreviations

GBD: GTPase binding domain
GFP: green fluorescent protein
TIRF: total internal reflection fluorescence
YFP: yellow fluorescent protein
