# Supplementary material for: An Actin-Based Wave Generator Organizes Cell Motility
Source: PLoS Biol. 2007 Aug 14;5(9):e221. doi: 10.1371/journal.pbio.0050221 (PMC1945041; doi:10.1371/journal.pbio.0050221)

Supplemental Figure S1

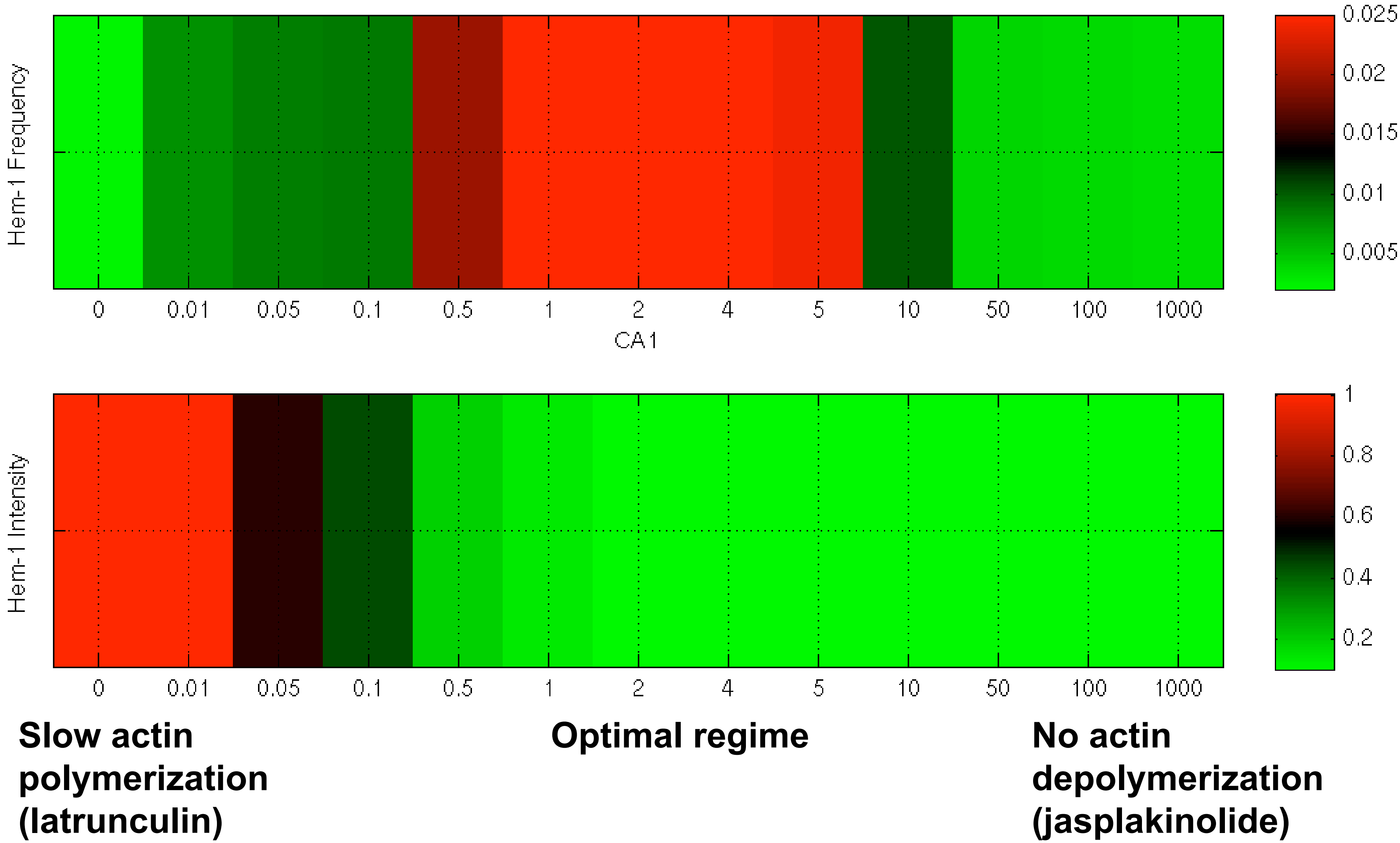

Supplement: Supplementary file 1 [file pbio.0050221.sg001.pdf]
