# Supplementary material for: An Actin-Based Wave Generator Organizes Cell Motility
Source: PLoS Biol. 2007 Aug 14;5(9):e221. doi: 10.1371/journal.pbio.0050221 (PMC1945041; doi:10.1371/journal.pbio.0050221)
Supplement: Supplementary file 2 [file pbio.0050221.sd001.doc]

```
function Hem1_v12(param)
% -------------------------------------------------------------------------
% Code: Steve Altschuler, Lani Wu, Orion Weiner
% Date: Jan 20, 2007
%
% Model:

% -------------------------------------------------------------------------

% 1) Call RunSimulation with range of constants
c_A1 = [1];
c_H2 = [.05];
for i = 1:length(c_A1)
    for j = 1:length(c_H2)
        RunSimulation(c_A1(i),c_H2(j));
    end
end

% --------------------------------------------------------------------------
function RunSimulation(c_A1,c_H2)

% 1) get simulation parameters
param = GetParam;
% change param struct for scan through constants
param.c_H    = [c_A1 c_H2];
param.file = [param.file '_cA1_' num2filestr(c_A1) '_cH2_' num2filestr(c_H2)];

for t = 1:param.T
    [c_A1 c_H2 t]
    % 2) set up information for ODE solver
    [T Y_0] = GetODEParams(param);

    % 3) call ODE solver
    [T,Y] = ode45(@evolvesystem,T,Y_0,[],param);

    % unpack Y
    sz = size(param.H);
    param.A = reshape(Y(end,1:end/2),sz);
    param.H = reshape(Y(end,end/2+1:end),sz);
    param.T_current = t*param.dt;

    % 4) update Hem
    param = UpdateHem(param);

    % 5) drug effects
    switch param.drug.type
        case 'Lat' % remove actin everywhere
            if param.T_current>param.drug.T
                param.A = zeros(size(param.A));
                param.drug.fNodrug = 0;
            end
        case 'Jas'  % stabilize actin where ever hem is nucleated
            if param.T_current>param.drug.T
                param.A = 1.0*((param.A>0)|param.HState);
                param.drug.fNodrug = 0;
            end
    end

    % 6) plot/store results
    results.HState(:,:,t) = param.HState;
    results.A(:,:,t) = param.A;
    results.H(:,:,t) = param.H;
end

% 6) save parameters and make movie
if ~strcmp(param.file,'')
    save(param.file,'param');
    save([param.file '_results'], 'results');
    MakeMovie(param.file, results)
end

% --------------------------------------------------------------------------
function param = UpdateHem(param)
% 1) Change from state 0 to 1
H_C = max(0,(param.HTotal - sum(sum(param.H)))/param.HTotal);
kSA = conv2(param.H,param.G_SA,'same');
lambda = H_C*param.dt*kSA;

Q = poissrnd(lambda,size(param.H));
mk1 = (param.HState == 0) & Q & (param.RegionMask);

% 2) Change from state 1 to 0
mk0 = (param.HState == 1) & (param.H < param.H_thresh_off) & (param.RegionMask);

% 3) Update
% reset param values
param.H(mk0) = 0;
param.H(mk1) = param.H_thresh_on;
if param.drug.fNodrug ==1
    param.A(mk0) = 0;
end

% reset states
param.HState(mk0) = 0;
param.HState(mk1) = 1;

% --------------------------------------------------------------------------
function [T Y_0] = GetODEParams(param)
T   = [0 param.dt];
sz  = size(param.A);
Y_0 = [reshape(param.A,sz(1)*sz(2),1); reshape(param.H,sz(1)*sz(2),1)];

% --------------------------------------------------------------------------

function dydt = evolvesystem(t,y,param)
% 0) unpack y = [A..H..]
L = length(y)/2;
[A H] = Unpack(L, y);

% 1) compute Short range Activator kernel
kSA = conv2(H,param.G_SA,'same');

% 2) compute cytosolic hem pool
H_C = (param.HTotal - sum(sum(H)))/param.HTotal;

% 3) update differentials
dA = param.c_A(1)*H.*(1-A).*param.RegionMask;
dH = param.c_H(1)*((1-A).*H_C.*kSA - param.c_H(2)*A.*H).*param.RegionMask;

% 4) non-nucleated hem sites don't evolve A or H
dA = dA.*param.HState;
dH = dH.*param.HState;

% 5) take care of drug effect (fNodrug = 0 if drug, = 1 if no drug)
dA = param.drug.fNodrug * dA;

% 6) repack dY = [dA..dH..]
dydt = [reshape(dA,L,1); reshape(dH,L,1)];

% --------------------------------------------------------------------------
function param = GetParam
% 0) Make simulations deterministic
param.state = 136;
rand('state', param.state);
randn('state', param.state);

% 1) Domain parameters
% T:  total simulation time T
% W:  width W of disk domain
% dt: H nucleation updated every time step dt;
%     A, H, R updated continuously in [t,t+dt]
param.T  = 500;
param.dt = 1;
param.T_current = 0;
param.W = 50;
param.N = 2*param.W +1;

% disk domain & boundary for simulation in square grid
param = BuildDomainInformation(param);

% 2) Model parameters
%  (a) Kernel [G_SA] = Short range Activator
%   sigma: steepness of Gaussian, radius: size of kernel window.
%   radius = 0: Gaussian assumed so wide that it's constant = 1/(area disk)
param.kernel.sigma  = [1];
param.kernel.radius = [1];
param.kernel.width  = 2 * param.kernel.radius +1;
param = BuildKernels(param);

%  (b) evolution equation constants
%   d/dt A = c_A(1)*[(1-A)H]
%   d/dt H = c_H(1)*[(1-A)(H_c)(H*G_SA) - c_H(2)(H)(A)]

param.c_A  = [1];
%param.c_H  = [1 1];
param.HTotal = 1000;
param.H_thresh_on  = 0.1;
param.H_thresh_off = 0.01;

% 3) Initialize structures
param.A_0  = zeros(param.N);
param.H_0  = zeros(param.N);
param.A    = zeros(param.N);
param.H    = zeros(param.N);

% state 0=off 1=on
param.HState = zeros(param.N);

% 4) Set up initial conditions
cInitCond = 0;
switch cInitCond
    case 0
        % point source
        param.A_0(param.W-1:param.W+1,param.W-1:param.W+1) = 0.1;
        param.H_0(param.W-1:param.W+1,param.W-1:param.W+1) = 0.5;
        param.HState(param.W-1:param.W+3,param.W-1:param.W+3) = 1;
        param.file = 'pointstart';
    case 1
        % hem in random state on disk of radius r_d to start
        mk = (rand(size(param.R_0)) >= 0.95) & param.RegionMask;
        r_d = 30;
        mk_d = getnhood(strel('disk',r_d,0));
        mk_init = zeros(size(mk));
        I = param.W+1-r_d:param.W+1+r_d;
        mk_init(I,I) = mk_d;
        mk = mk & mk_init;
        param.HState(mk) = 1;
        param.file = 'results/fieldstart';
end
param.version = 'Hem1_v11.m';
param.file = [param.file '_' datestr(now,'yyyymmddTHHMMSS')];
param.A = param.A_0;
param.H = param.H_0;
% 5) Drugs
param.drug.type = ''; %'Jas'; %'Lat' % ''
param.drug.T = 10;
param.drug.fNodrug = 1; % initially no drug

% --------------------------------------------------------------------------
% --------------------------------------------------------------------------
function MakeMovie(mfile, results)
h = figure
set(gcf, 'position',[11 42 425 941]);
try % close avi file if problem
    aviobj = avifile(mfile,'compression', 'none', 'fps', 20)
    sz = size(results.A);
    for t = 1:sz(3)
        PlotResults(results,t);
        frame = getframe(gcf);
        aviobj = addframe(aviobj,frame);
    end
    aviobj = close(aviobj);
catch
    aviobj = close(aviobj);
end
close(h)
% -------------------------------------------------------------------------
function PlotResults(results,t)
% plot results of simulation
sz = size(results.A);

subplot(2,1,1)
imagesc(results.A(:, end:-1:1,t)',[0 1])
c = colormap('Gray');
colormap(c(end:-1:1,:))
colorbar
SetFrameSize(sz(1));
title(num2str(t))

subplot(2,1,2)
imagesc(results.H(:, end:-1:1,t)',[0 1])
title('H')
colorbar

SetFrameSize(sz(1));

%--------------------------------------------------------------------------
function [M] = SetFrameSize(N)
axis([0 N 0 N])
axis square
grid off

% -------------------------------------------------------------------------
function param = BuildKernels(param)
% build kernals G_SA,G_MA,G_LI

param.G_SA = fspecial('gaussian',param.kernel.width(1),param.kernel.sigma(1));

% --------------------------------------------------------------------------
function [H_C] = ComputeCytoplasmicHemFraction(HTotal, H)
H_C = (HTotal - sum(sum(H)))/HTotal;
if H_C < 0
    H_C = 0;
    'H_C<0'
end

% -------------------------------------------------------------------------
function param = BuildDomainInformation(param)
% define regions and region properties for simulation

param.RegionMask = getnhood(strel('disk',param.W,0));
[B,L] = bwboundaries(param.RegionMask,'noholes');
param.RegionMaskBd = B{1};
param.RegionArea = sum(sum(param.RegionMask));

% --------------------------------------------------------------------------
function [A H] = Unpack(L,y)
sz_square = [sqrt(L) sqrt(L)];
A = reshape(y(1:L),sz_square);
H = reshape(y(L+1:2*L),sz_square);

%--------------------------------------------------------------------------
function s = num2filestr(n)
s = num2str(n);
I = findstr(s,'.');
s(I) = '-';
```
